# Adherence to Iron and Folic Acid Supplementation and its determinants among pregnant women in East Africa: Analysis of Demographic and Health Surveys data from nine East African countries

**DOI:** 10.1371/journal.pone.0327410

**Published:** 2025-07-16

**Authors:** Nanati Legese Alemu, Kedir Teji Roba, Henok Demeke Getaneh, Temam Beshir Raru, Alemneh Kabeta Daba, Netsanet Worku, Zeweter Abebe

**Affiliations:** 1 College of Health and Medical Sciences, Haramaya University, Harar, Ethiopia; 2 The Institute for Mental and Physical Health and Clinical Translation (IMPACT), Deakin University, School of Medicine, Barwon Health, Geelong, Victoria, Australia; 3 College of Medicine and Health Sciences, Hawassa University, Hawassa, Ethiopia; 4 Institute of Public Health, University of Gondar, Gondar, Ethiopia; 5 College of Natural Sciences, Addis Ababa University, Addis Ababa, Ethiopia; PLOS ONE, UNITED KINGDOM OF GREAT BRITAIN AND NORTHERN IRELAND

## Abstract

**Introduction:**

Anemia due to deficiency of nutrients like iron and folic acid (IFA) is one of the global public health issues that has contributed to an unacceptably high proportion of maternal and childhood morbidity and mortality. IFA supplementation (IFAS) during pregnancy is critical for reducing anemia and related undesired outcomes. However, comprehensive evidence on the magnitude of adherence to IFA supplementation and its associated factors in East Africa remains limited.

**Objective:**

The objective of this study was to assess the level of adherence to IFAS during pregnancy and to identify the factors associated with adherence among pregnant women in East African countries.

**Methods:**

In this study, we analyzed demographic and health survey (DHS) data from nine countries in East Africa, yielding a sample of 57,283 pregnant women. The study used multilevel mixed effects cross-sectional design. We applied four models, and we compared the models using the Akaike information criterion (AIC) and the Bayes information criterion (BIC). The model with smaller AIC and BIC was the best to fit the data, and the interpretation of the fixed effects was based on this model. To measure cluster variation, we used the intra-cluster correlation coefficient (ICC) standard deviation. Finally, we utilized fixed effects to estimate the association between adherence to IFAS and the independent variables, and we reported the results as an odds ratio with a 95% confidence range.

**Results:**

The overall prevalence of adherence to IFAS among pregnant women in East Africa was 35.8% (95% CI: 35.4–36.2). This prevalence ranges from 3.8% in Burundi to 83.7% in Zambia, with significant differences between countries. Adherence to IFAS among women with secondary and more than secondary level of education was higher by 29% (AOR = 1.29, 95% CI: 1.19, 1.41) and 92% (AOR = 1.92, 95% CI: 1.68, 2.20), respectively, compared to women with no formal education. Adherence to IFAS among women who had the first ANC visit on the second and third trimesters was lower by 16% (AOR = 0.84, 95% CI: 0.80, 0.89) and 74% (AOR = 0.26, 95% CI: 0.23, 0.30), respectively, compared to those with the first ANC visit on the first trimester. Furthermore, four or more ANC visits during pregnancy and a lower distance to health facilities were significantly associated with adherence to IFAS in East Africa.

**Conclusions:**

Only one-third of pregnant women in East Africa adhered to IFAS. Adherence was significantly associated with higher education, early ANC booking, more frequent ANC visits, proximity to health facilities, and country of residence. Interventions should prioritize improving women’s education, promoting timely and frequent ANC attendance, and addressing country-specific barriers to improve IFAS uptake and maternal outcomes.

## Introduction

To reduce the risk of low birth weight, maternal anemia, and iron deficiency, the WHO recommends that all pregnant women begin receiving a regular dose of 400 µg (0.4 mg) of folic acid and 30–60 mg of iron as soon as possible during the first trimester of pregnancy and continue throughout the pregnancy. A higher dose is recommended in areas where anemia in pregnant women is a serious public health problem [1,2]. According to the WHO’s operational guidance, adherence to IFAS during pregnancy is commonly measured by the number of days the supplement is consumed. A widely accepted threshold, used in large-scale surveys like DHS and Multiple Indicator Cluster Surveys (MICS), is the consumption of supplements for at least 90 days during pregnancy, regardless of whether those days are consecutive. While the WHO originally defined full adherence as taking for at least 6 months in accordance with national protocols, the 90-day threshold is widely adopted for monitoring and evaluation to report the proportion of women achieving minimal adherence [3].

Iron and folic acid (IFA) deficiencies during pregnancy have a significant impact on the pregnant women’s health, her pregnancy, and fetal development [2,4]. Maternal anemia during pregnancy has contributed to low maternal weight gain, congestive heart failure, preterm labor, hemorrhage, decreased resistance to infection, and impaired cognitive development of the fetus [5]. Folic acid shortage during conception and early pregnancy has also been linked to an increased risk of premature delivery and birth asphyxia, low birth weight, puerperal infection, preterm birth, birth abnormalities or neural tube disorders, fetal anomalies, and newborn mortality [6–9]. The prevalence of these risks is higher in low- and middle-income nations [10]. To avoid the risks, pregnant women are advised to take the recommended IFAS pills during pregnancy and/or postpartum [1,11,12]. However, globally, nearly three-fourths (70%) of pregnant women do not take daily IFAS as recommended [13], and only 6 out of 22 Sub-Saharan countries have reported adherence rates above 50% [3].

Although a previous study using DHS data from Sub-Saharan African countries was conducted, its coverage of East African nations was outdated, relying on data collected between 2013 and 2015 [13]. In our study, we excluded Kenya’s DHS data due to its pre-2015 collection and incorporated more recent datasets from Madagascar, Rwanda, and Zambia. Furthermore, unlike the earlier study, we applied multilevel logistic regression models to account for clustering within higher-level sampling units, thereby improving the accuracy of our estimates [14].

In addition, several localized studies have assessed adherence to IFAS during pregnancy [15–17], and one systematic review addressed the topic; however, it included only studies from Ethiopia, India, Nepal, and Nigeria [18,19]. Despite these efforts, there is still a lack of up-to-date and comprehensive data on IFAS adherence specifically in Eastern Africa.

Thus, this study will use the most recent DHS data from nine East African nations (Burundi, Ethiopia, Madagascar, Malawi, Rwanda, Tanzania, Uganda, Zambia, and Zimbabwe) to evaluate the prevalence and drivers of IFAS adherence among women in eastern Africa. The results contribute to our understanding of local successes and factors that facilitate or impede best practices.

## Materials and methods

### Study setting and data source

The United Nations divided Africa into five regions. East Africa is the largest region, with 19 countries (Burundi, Comoros, Djibouti, Ethiopia, Eritrea, Kenya, Madagascar, Malawi, Mauritius, Mozambique, Reunion, Rwanda, Seychelles, Somalia, Somaliland, Tanzania, Uganda, Zambia, and Zimbabwe). We used DHS data from East African countries in this analysis. DHS is a nationally representative household survey that uses face-to-face interviews of women aged 15–49 to collect data from a wide range of demographic, health, and nutrition tracking and effect evaluation measures. The survey employs stratified, multi-stage, random sampling. Detailed survey techniques and sampling methods used to collect data have been documented elsewhere [20].

To gather data that is similar between nations, the DHS program uses standardized techniques such as uniform field procedures, manuals, and questionnaires, and it is usually conducted every five years [20].

Among East African countries, DHS was not conducted in Djibouti, Somalia, Somaliland, the Seychelles and Mauritius, or Reunion, while it was conducted before 2015 in Eritrea, Comoros, Kenya, and Mozambique.

In this study, we included the recent DHSs from 9 East African countries (Burundi, Ethiopia, Madagascar, Malawi, Rwanda, Tanzania, Uganda, Zambia, and Zimbabwe). We limited the DHS analysis to nine East African countries because it was not conducted in six of those countries, and we included survey data after 2015 because, earlier DHS data are considered outdated. Table 1 shows the sample size and study period of each country.

**Table 1:** The study participants of adherence to IFAS and its determinants among pregnant women in East African countries, n = 57,283.

Country	Year of Study	Unweighted sample size (%)	Weighted sample size (%)
Burundi	2016−17	4,091(7.1)	4257(7.4)
Ethiopia	2016	3,266(5.7)	3192(5.6)
Madagascar	2021	6,769(11.8)	6865(11.9)
Malawi	2015−16	12,167(21.2)	12077(12.0)
Rwanda	2019−20	4,997(8.7)	5081(8.8)
Tanzania	2015−16	5,675(9.9)	5741(10.0)
Uganda	2016	9,113(15.9)	8977(15.6)
Zambia	2018	7,147(12.5)	7128(12.4)
Zimbabwe	2015	4,058(7.1)	4156(7.2)

We obtained the DHS data from the official database of the DHS program, www.measuredhs.com, after we got authorization through an online request by stating the purpose of the study. The dependent and independent variables for this study were taken from the Kids Recode (KR file) data set. The KR dataset has a record for every child of interviewed women, born in the five years preceding the survey. It contains information related to the child’s pregnancy, post-natal care, immunization, and health. The source population consisted of all pregnant women aged 15–49 years who had IFAS data. The study cohort consisted of pregnant women aged 15–49 years who were given or bought iron tablets/syrup during pregnancy.

### Variables

The outcome variable for this study was IFAS consumption for 90 days or more, which was dichotomized as “IFAS consumption for less than 90 days = 0, IFAS consumption for 90 or more days = 1. This 90-day threshold, aligned with WHO operational guidance, providing a practical metric based on DHS data [3].

We examined independent variables at the individual and community levels in this study. Country and residency were the two community-level factors. The variables measured at the individual level included maternal age, wealth status, marital status, education level of both the woman and her husband, mode of last delivery, history of pregnancy termination, family size, gravidity, media exposure, ANC follow-up status, and distance to the health facility. We have listed the variable’s description below.

#### Wealth index.

The wealth index is a composite measure of a household’s cumulative living standard. In the DHS, households are given wealth index scores based on the number and kinds of consumer goods they own, ranging from a television to a bicycle or car, in addition to housing characteristics such as the source of drinking water, toilet facilities, and flooring materials. These scores are derived using principal component analysis. National wealth quintiles are compiled by assigning the household score to each usual household member, ranking each person in the household population by her or his score, and then dividing the distribution into five equal categories (poorest, poorer, middle, richer, and richest) [21]. Based on previous literature, we re-categorized the wealth index from 5 quintiles into 3 categories by combining poorest and poorer into one category (called “poor”); middle wealth level into the second category (called “middle”); and richer and richest into the third category (called “rich”) [13,22].

#### Education level attended.

This variable indicates the level of education in the following categories: none, primary, secondary, and more than secondary.

#### Family size.

We classified this variable into groups of lower than five and greater than five and more to examine how big family sizes affect the use of IFAS. Our decision to employ this classification was supported by earlier research. “A large family is defined as a group of people who live together in a dwelling unit and have five or more members, or three or more children, whether they are related by blood or marriage” [23].

#### First antenatal care (ANC) booking.

In DHS data, the timing of the first antenatal visit is grouped into categories of “no antenatal visit,” “<= 3 months,” “4-5 months,” “6-7 months,” “8+ months,” and “missing/don’t know” before calculating percentages. The percentages are the quotients of the numerators divided by the denominator, multiplied by 100 [24]. For our analysis, we reclassified these categories into trimesters: first trimester (≤3 months), second trimester (4–6 months), and third trimester (≥7 months).

#### Number of ANC follow-up.

The number of ANC visits in DHS data was grouped into categories of “no antenatal care,” “1 visit,” “2-3 visits,” “4+ visits,” and missing/don’t know” [24]. The denominator for the percentage calculation was the number of women who gave birth in the last 5 years. Based on a thorough literature review, the authors reclassified this variable into two categories: fewer than four visits and four or more visits [1].

#### Ever had terminated pregnancy.

This variable is if the respondent ever had a pregnancy that terminated in a miscarriage, abortion, or stillbirth (a pregnancy that did not result in a live birth) [24].

#### Last birth a cesarean section.

This variable indicates if the last child was born by cesarean section. The denominator of this variable is those respondents who have had one or more births in the five years preceding the survey [24].

#### Media exposure.

This variable was assessed by examining the experience of watching television, listening to the radio, and reading newspapers at least once a week. We created a new variable called “media exposure” by merging three mass media (TV, radio, and newspapers). The variable media exposure was labeled as “yes” if the respondent had experience with at least one of the three mass media channels and “no” if they had no experience with any of the three mass media.

### Data processing and management

STATA software (StataCorp. 2015. Stata Statistical Software: Release 14. College Station, TX: StataCorp LP) was used for data processing and analysis. Before any statistical analysis, the data were weighted using sampling weight, primary sampling unit, and strata to restore the survey’s representativeness and to instruct STATA to take the sampling design into account when calculating standard errors to provide reliable statistical estimates. To describe the study population, cross-tabulations and summary statistics were used.

Because the DHS data is hierarchical, women within a cluster may be more similar to each other than women in the other cluster. As a result, the principle of independent observations and equal variance across clusters may be violated. To obtain a credible standard error and unbiased estimate, we used a sophisticated statistical model (multilevel regression model) that accounts for between-cluster variability.

The bi-variable multilevel mixed-effect logistic regression model was fitted for all independent variables, and the variables with a P-value of 0.25 in the bi-variable multilevel mixed-effects logistic regression analysis were candidates for the multi-variable mixed-effects logistic regression. A mixed-effect model with the lowest AIC and BIC was selected. Variables having a p-value of 0.05 were judged as significant drivers of IFA use in the multivariable multilevel mixed-effect analysis. To measure cluster variation, the intra-cluster correlation coefficient (ICC) standard deviation was used. Besides, the model comparison was done based on the AIC and BIC.

We applied four different models to this study analysis. Model 1 was a null model (it did not include exposure variables) and was used to validate community variance and to give evidence to assess random effects at the community level. The second model was a multivariable model (adjusted for individual-level variables), and in the third model, we included community-level factors. In model 4, we included potential candidate factors from both the individual and community levels. We used fixed effects (a measure of association) to estimate the relationship between IFAS adherence and independent variables, and we reported as an odds ratio with a 95% confidence range.

## Results

### Background characteristics of respondents and adherence to IFAS

Out of all women (57,283) who gave birth in the five years preceding the survey, the majority of study participants (44.6%) were between the ages of 25 and 35, with 77.1% being rural residents and 53.5% having a primary level of education. The majority of respondents (82.2%) were married women, and 53.9% had no media exposure (Table 2).

**Table 2:** Weighted socio-demographic characteristics of study participants, n = 57,283.

Characteristics	Unweighted	Weighted frequency (%)	Adherence to IFA	
			Yes (%)	No (%)
**Age**				
Less than 25 years	18,431(32.2)	18598(32.4)	7139(38.4)	11459 (61.6)
25–35 years	25,475(44.5)	25644(44.6)	9081(35.4)	16563(64.6)
More than 35 years	13,377(23.4)	13231(23.0)	4360(33.0)	8870(67.0)
**Residence**				
Urban	13948(24.4)	13146 (22.9)	5889(44.8)	7257(55.2)
Rural	43335(75.7)	44327 (77.1)	14692(33.1)	29635(66.9)
**Country**				
Burundi	4091(7.1)	4257(7.4)	159(3.8)	4,097(96.2)
Ethiopia	3266(5.7)	3192(5.6)	480(15.0)	2,711(85.0)
Madagascar	6769(11.8)	6865(11.9)	2019(29.4)	4,844(70.6)
Malawi	12167(21.2)	12077(21.0)	4,702(38.9)	7,374(61.1)
Rwanda	4997(8.7)	5081(8.8)	1014(20.0)	4066(80.0)
Tanzania	5675(9.9)	5741(10.0)	1,629(28.4)	4,111(71.6)
Uganda	9113(15.9)	8977(15.9)	2,507(27.9)	6,469(72.1)
Zambia	7147(12.5)	7128(12.4)	5,966(83.7)	1,161(16.3)
Zimbabwe	4058(7.1)	4156(7.2)	2,100(50.6)	2,055(49.4)
**Education level**				
No formal education	9292(16.2)	9242(16.1)	2097(22.7)	714577.3
Primary	30490(53.2)	30743(53.5)	10449(34.0)	20294(66.0)
Secondary	15323(26.8)	15325(26.7)	6927(45.2)	8398(54.8)
More than secondary	2178(3.8)	2163(3.8)	1109(51.2)	1055(48.8)
**Husband Education level (n = 46,975)**				
No formal education	6217(13.2)	6254(13.2)	1357(21.7)	4897 (78.3)
Primary	22702(48.3)	23156(49.0)	7158(30.9)	15998(69.1)
Secondary	14001(29.8)	13979(29.6)	6273(44.9)	7706(55.1)
Higher	3261(6.9)	3080(6.5)	1437(46.7)	1642(53.3)
Don’t know	794(1.7)	796(1.7)	361(45.4)	434(54.6)
**Wealth status**				
Poor	23996(41.9)	23711(41.3)	8044(33.9)	15666(66.1)
Middle	10786(18.8)	11091(19.3)	3838(34.6)	7254(65.4)
Rich	22501(39.3)	22671(39.5)	8699(38.4)	13972(61.6)
**Marital status**				
Single/Divorced/widowed/ separated	10,308(18.0)	10209(17.8)	3995(39.1)	6214(60.9)
Married/living with partner	46,975(82.0)	47264(82.2)	16586(35.1)	30678(64.9)
**Family size**				
< 5	20271(35.4)	20701(36.0)	7575(36.6)	13126(63.4)
>=5	37012(64.6)	36772(64.0)	13006(35.4)	23766(64.6)
**Sex of household head**				
Male	43670(76.2)	44147(76.8)	15655(35.5)	28492(64.5)
Female	13613(23.8)	13326(23.2)	4926(37.0)	8400(63.0)
**Media exposure**				
Yes	26405(46.1)	26499(46.1)	10055(37.9)	16444(62.1)
No	30878(53.9)	30974(53.9)	10526(34.0)	20448(66.0)

### Weighted reproductive characteristics of study participants

In this study, 98.5% of participants received prenatal care during their pregnancy. More than half of them (58.3%) scheduled their first prenatal care checkup during the second trimester of pregnancy. Approximately 40.5%, 40.0%, and 14.0% had less than four antenatal visits, lived far from health facilities, and had a history of abortion, respectively (Table 3).

**Table 3:** Reproductive characteristics of study participants, in East Africa, n = 57,283.

Characteristics	Un-weighted	Weighted	Adherence to IFA	
**Yes (%)**	**No (%)**
**ANC follow-up**				
Yes	56527(98.7)	56607(98.5)	20354(36.0)	36254(64.0)
No	756(1.3)	866(1.5)	227(26.3)	638(73.7)
**First ANC booking**				
First trimester	20517(36.2)	20293(35.7)	8079(39.8)	12214(60.2)
Second trimester	32935(58.8)	33119(58.3)	11912(36.0)	21207(64.0)
Third trimester	3306(5.8)	3438(6.1)	501(14.6)	2936(85.4)
**Number of ANC follow‑up**				
Less than 4 visits	23074(40.3)	23293(40.5)	6019(25.8)	17275(74.2)
>=4 visits	34209(59.7)	34180(59.5)	14563(42.6)	19617(57.4)
**Total children ever born (gravidity)**				
< 5	41855(73.1)	42307(73.6)	15794(37.3)	26513(62.7)
5+	15428(26.9)	15166(26.4)	4787(31.6)	10379(68.4)
**Recent delivery with C-section**				
Yes	4063(7.1)	4033(7.0)	1625(40.3)	2409(59.7)
No	53074(92.9)	53297(93.0)	18915(35.5)	34382(64.5)
**Distance to health facilities**				
Big problem	22450(39.2)	22992(40.0)	7728(33.6)	15264(66.4)
Not big problem	34833(60.8)	34481(60.0)	12853(37.3)	21628(62.7)
**Ever had terminated pregnancy**				
Yes	8056(14.1)	8056(14.0)	2602(32.3)	5454(67.7)
No	49227(85.9)	49417(86.0)	17979(36.4)	31438(63.6)
**Pooled prevalence**			35.8% (95% CI: 35.4–36.2)	64.2% (95% CI: 63.8–64.6)

### Adherence to IFA supplementation

According to this study, the prevalence of IFAS adherence was 35.8% (95% CI: 35.4–36.2). This prevalence ranges from 3.8% in Burundi to 83.7% in Zambia, indicating significant differences between countries. Among the total pregnant women who scheduled their first ANC booking in the first trimester (35.7%) and second trimester (58.3%), nearly 40% and 36% were adherent to IFASs, respectively. On the other hand, among pregnant women who scheduled their first ANC booking in the third trimester (6.1%), 14.6% were adherent to IFASs (Table 3).

### Factors associated with adherence to IFA supplementation during pregnancy

#### The result of the random effects.

The null model results revealed that there was statistically significant variability in the odds of adherence to IFAS with a community variance of 2.157. And the ICC in the null model indicated that 39.61% of the total variability in the adherence was ascribed to the differences between the communities. In model 4, community variance (0.62) and standard error (0.006) remained significant but were reduced, and 16.04% of the total variance of adherence to IFAS can be ascribed to the community (Table 4).

**Table 4:** Multi-variable multilevel logistic regression analysis of factors associated with adherence to IFAS in East Africa.

Variables	Models			
	Model 1 AOR (95%CI)	Model 2 AOR (95%CI)	Model 3 AOR (95%CI)	Model 4 AOR (95%CI)
**Age of respondent**				
Less than 25 years	**–**	1.00	**–**	1.00
25–35 years	**–**	0.95(0.90, 1.00)	**–**	0.98 (0.93, 1.03)
More than 35 years	**–**	0.95(0.88, 1.02)	**–**	1.00 (0.92, 1.07)
**Marital status**				
Single/Divorced/widowed/separated	**–**	1.00	–	1.00
Married/living with partner	**–**	0.98(0.92, 1.05)	–	1.02 (0.96, 1.09)
**Education Level**				
No formal education	**–**	1.00		1.00
Primary	**–**	1.30(1.21, 1.40)**	**–**	1.07 (0.99, 1.15)
Secondary	**–**	1.76(1.61, 1.92)**	**–**	1.29 (1.19, 1.41)**
More than secondary	**–**	2.61(2.27, 2.99)**	**–**	1.92(1.68, 2.20)**
**Media exposure**				
No	**–**	1.00	**–**	1.00
Yes	**–**	0.98(0.93, 1.03)		0.97 (0.93, 1.02)
**Wealth status**				
Poor	**–**	1.00		1.00
Middle	**–**	0.98(0.92, 1.04)	**–**	1.02 (0.96, 1.08)
Rich	**–**	0.97(0.91, 1.04)	**–**	1.06(0.99, 1.13)
**Sex of household head**				
Male	**–**	1.00	**–**	1.00
Female	**–**	0.99(0.93, 1.05)	**–**	1.00 (0.94, 1.06)
**Gravidity**				
< 5	**–**	1.00	**–**	1.00
>=5	**–**	1.00(0.94, 1.07)	**–**	0.94(0.88, 1.00)
**First ANC booking**				
First trimester	**–**	1.00		1.00
Second trimester	**–**	0.91(0.87, 0.96)**	**–**	0.84(0.80, 0.89)**
Third trimester	**–**	0.29(0.26, 0.33)**	**–**	0.26(0.23, 0.30)**
**Number of ANC follow‑up**				
Less than 4 visits	**–**	1.00		1.00
>=4 visits	**–**	1.87(1.78, 1.96)**	**–**	1.79(1.70, 1.88)**
**Distance to health facilities**				
Big problem	**–**	1.00	**–**	1.00
Not big problem	**–**	1.09(1.03, 1.14)**	**–**	1.11(1.05, 1.16)**
**Ever had terminated** **pregnancy**				
No	**–**	1.00	**–**	1.00
Yes	**–**	0.99(0.93, 1.05)		1.03 (0.96, 1.09)
**Country**				
Ethiopia	**–**	–	1.00	1.00
Burundi	**–**	–	0.22(0.18, 0.27)**	0.18(0.14, 0.23)**
Madagascar			2,33(2.0, 2.61) **	2.00(1.69, 2.38)**
Malawi	**–**	–	3.74(3.35, 4.17)**	4.37(3.70, 5.15)**
Rwanda	**–**	–	1.43(1.27, 1.62)**	1.28(1.07, 1.54) **
Tanzania	**–**	–	2.22(1.97, 2.50)**	2.40(2.01, 2.86) **
Uganda	**–**	–	2.14(1.91, 2.39)**	2.05(1.73, 2.43)**
Zambia	**–**	–	30.7(27.2, 34.6)**	39.2(32.7, 47.0)**
Zimbabwe			5.79(5.14, 6.53)**	5.49(4.55, 6.62)**
**Place of residence**				
Urban	**–**	–	1.00	1.00
Rural	**–**	–	0.82(0.77, 0.85)**	0.95(0.87, 1.04)
**Random effects**				
Community variance	2.157 (0.07)	1.99 (0.07)	0.66 (0.003)	0.62 (0.006)
ICC%	39.61%	37.7%	16.8%	16.04%
**Model comparison**				
AIC	64511.82	**62086.24**	60693.11	**58371.96**
BIC	64529.73	62247.28	60791.62	**58613.52**

**, Significant at P-value < 0.05; ICC, intra-class correlation coefficient; AOR, adjusted odds ratio; AIC, Akaike information criteria; BIC, Bayesian information criteria.

#### The results of the fixed effects.

The model with smaller AIC and BIC was the best to fit the data, and the interpretation of the fixed effects was based on this model. Model-4 was adjusted for both individual and community-level factors and has a smaller AIC and BIC compared to other models. Lower AIC and BIC values suggest models that have a better fit to the data while avoiding over fitting.

Based on our study, the odds of adherence to IFAS among women with secondary and higher than secondary levels of education were higher by 29% (AOR = 1.29, 95% CI: 1.19, 1.41) and 92% (AOR = 1.92, 95% CI: 1.68, 2.20), respectively, compared to women with no formal education. The odds of adherence to IFAS among women who had the first ANC visit on the second and third trimesters were lower by 16% (AOR = 0.84, 95% CI: 0.80, 0.89) and 74% (AOR = 0.26, 95% CI: 0.23, 0.30), respectively, compared to those who visited on the first trimester.

Adherence to IFAS was 79% higher (AOR = 1.79, 95% CI: 1.70, 1.88) on women who had more than four ANC visits compared to those who had less than four ANC visits. On the other hand, pregnant women who lived near healthcare facilities had 1.11 times (AOR = 1.11(1.05, 1.16)) higher odds of IFAS adherence than those who resided far away. Women in Zambia had a 39.19 (AOR = 39.19 (32.65, 47.03)) times higher adherence rate to IFAS than women in Ethiopia. However, women in Burundi had 82% lesser (AOR = 0.18, 95% CI: 0.14, 0.23) adherence to IFAS in contrast to women in Ethiopia (Table 4).

## Discussion

The current study assessed adherence to IFAS among pregnant women across nine East African countries and found an overall adherence prevalence of 35.8%. This rate is higher than the 28.7% overall prevalence of adherence using 22 Sub-Saharan African (SSA) nations’ DHS data [13]. The difference may be attributed to the more recent and regionally updated DHS data used in our analysis. The later used DHS data that was conducted before the year 2016.

We observed substantial variation between countries, with Zambia reporting the highest adherence at 83.7% and Burundi the lowest at 3.8%. Zambia’s adherence rate aligns with findings from Bangladesh [25] and Cambodia [26], where rates reached 80% and 75.9%, respectively. Zambia’s high adherence rate may be attributed to strong public health education, a supportive digital health infrastructure, and effective community engagement. A study reported that 97.4% of women in Zambia had received messages about IFA [27], highlighting the reach of health education efforts that likely boosted awareness and uptake. The Zambia Electronic Perinatal Record System (ZEPRS), implemented in public obstetric clinics, has improved antenatal care by standardizing service delivery and strengthening follow-up [28]. Furthermore, Zambia’s early adoption of the Scaling Up Nutrition (SUN) program in 2010 has enhanced community-level outreach and multisectoral collaboration to promote maternal nutrition [29]. These coordinated strategies likely contribute to Zambia’s exceptional performance compared to other countries in the region.

In contrast, poverty may be the reason for Burundi’s extremely low rate of adherence to IFAS, which is consistent with the earlier study [13]. Burundi’s gross domestic product was ranked very low in the world [30]. Although IFAS is provided at no cost in most public health facilities, poverty may still influence adherence through indirect pathways. For instance, economically disadvantaged women may be unable to afford transportation to health facilities, face opportunity costs related to clinic attendance, or lack the flexibility to attend repeated ANC visits [31–33]. These barriers persist even when the supplements themselves are free and may help explain why Burundi one of the world’s poorest countries with limited healthcare infrastructure has such low adherence rates [34,35].

Consistent with previous research [36,37], we found that women with more than secondary education had 92% higher odds of IFAS adherence compared to those with no formal education. Educated women are more likely to understand the risks of anemia, recognize the benefits of supplementation, and have improved access to healthcare services [38], including the ability to purchase IFAS when necessary [34].

In comparison to the first ANC booking during the first trimester, the odds of adherence to IFAS were 16% and 74% lower for ANC bookings during the second and third trimesters, respectively. Besides, our study found that an increased number of ANC follow-ups (more than four visits) were significantly associated with better adherence to IFAS. These findings are consistent with previous systematic reviews and meta-analyses [39], a study in Bangladesh [29], a study in low-middle-income countries of Asian, African, Latin American, and Caribbean nations [30], and studies in Ethiopia [36,37,40]. The reason for these results could be that women who began ANC early or had frequent follow-ups are more likely to obtain adequate counseling on the use of IFAS. It also permits checking the women’s hemoglobin level and the growth of the fetus, which ensures supplementation of IFA throughout the pregnancy period. Besides, interactions during antenatal sessions are also important in conveying the need for IFAS during the pregnancy period [41,42]. As a result, behavioral interventions are required to improve first-trimester attendance and the need for preventative ANC care. Additionally, information on the particular services should be given at every visit [42].

Our study also found that women who lived near a health facility were more likely to adhere to IFAS, with 11% higher odds compared to those who lived farther away. This finding aligns with previous studies in Ethiopia and India [43,44], reinforcing evidence that transportation barriers, long travel distances, and poor road infrastructure remain major challenges to accessing maternal health services. When health facilities are not easily accessible, women may be discouraged from initiating ANC or attending follow-up visits, thereby reducing their chances of receiving and consistently using IFAS [34,45]. Improving equitable physical access to health facilities is essential, and interventions aimed at enhancing maternal health outcomes should prioritize strategic facility placement and transportation support [46,47].

### Strengths and Limitations

Our analysis was based on data from nationally representative multi-country surveys, which is the strength of this study. However, because the study used a cross-sectional study design, the results do not establish a causal relationship between the outcome variable and independent variables. Moreover, because the study used secondary data for analysis, it was unable to account for additional predictors such as cultural factors and the standard of care (including availability of IFA supplements and culture).

## Conclusion

According to the findings of the study, only 35.8% of pregnant women in East African nations adhered to the IFAS. Key predictors of adherence to IFAS included early ANC booking, a higher number of ANC Visits, higher educational attainment, and closer proximity to a health facility. To improve adherence, targeted efforts should focus on promoting early ANC attendance, increasing the frequency of visits, and expanding access to health information. Interventions must also address geographic barriers by ensuring that distance to health facilities does not hinder IFAS use. The study also highlighted significant variation in adherence between countries: while adherence was very high in Zambia, it was notably lower in Burundi and Rwanda. When choosing appropriate policies and program interventions, concerned bodies should be urged to consider the experience of those nations with better performance. Overall, strengthening women’s education, improving access to IFAS, and facilitating cross-country learning could help reduce iron deficiency anemia and its associated adverse pregnancy outcomes across the region.
